# Nonylphenol aggravates non-alcoholic fatty liver disease in high sucrose-high fat diet-treated rats

**DOI:** 10.1038/s41598-018-21725-y

**Published:** 2018-02-19

**Authors:** Jie Yu, Xuesong Yang, Xuefeng Yang, Mengxue Yang, Pan Wang, Yu Yang, Jing Yang, Wenmei Li, Jie Xu

**Affiliations:** 1 School of Public Health, Zunyi Medical University, Zunyi, Guizhou 563003 P.R. China; 2 Department of Hospital Infection and Control, The First People’s Hospital of Guiyang city, Guiyang, Guizhou 550002 P.R. China; 3 Department of Gastrointestinal Surgery, The Affiliated Hospital of Zunyi Medical University, Zunyi, Guizhou 563003 P.R. China; 4 Department of Endocrinology, The Affiliated Hospital of Zunyi Medical University, Zunyi, Guizhou 563099 P.R. China; 5 Department of Nuclear Medicine, The Affiliated Hospital of Zunyi Medical University, Zunyi, Guizhou 563099 P.R. China

## Abstract

Exposure to environmental endocrine disruptors (EEDs) contributes to the pathogenesis of many metabolic disorders. Here, we have analyzed the effect of the EED-nonylphenol (NP) on the promotion of non-alcoholic fatty liver disease (NAFLD) in rats fed high sucrose-high fat diet (HSHFD). Fifty Sprague-Dawley rats were divided into five groups: controls fed a normal diet (C-ND); HSHFD-fed controls (C-HSHFD); and rats fed a HSHFD combined with NP at doses of 0.02 μg/kg/day (NP-L-HSHFD), 0.2 μg/kg/day (NP-M-HSHFD), and 2 μg/kg/day (NP-H-HSHFD). Subchronic exposure to NP coupled with HSHFD increased daily water and food intake (*p* < 0.05), hepatic echogenicity and oblique liver diameter (*p* < 0.05), and plasma levels of alanine aminotransferase, aspartate aminotransferase, total cholesterol, triglycerides, and low density lipoprotein cholesterol (*p* < 0.05). Combined exposure to NP and HSHFD induced macrovesicular steatosis with dilation and congestion of the central vein, liver inflammatory cell infiltration, and expression of genes regulating lipid metabolism, SREBP-1C, FAS, and Ucp2. These results demonstrate that NP aggravates NAFLD in HSHFD-treated rats by up-regulating lipogenic genes, and that HSHFD increases the toxic effects of NP. Thus subchronic NP exposure may lead to NAFLD, especially when combined with a high-sucrose/high-fat diet.

## Introduction

Non-alcoholic fatty liver diseases (NAFLD) encompass a wide range of liver pathologies from simple steatosis to steatohepatitis and cirrhosis^1^, which may result in hepatocellular carcinoma. NAFLD is frequently associated with insulin resistance, dyslipidemia, obesity, and the metabolic syndrome^2^. Following the epidemics of obesity, NAFLD is emerging as an important public health issue in the world. The highest prevalence of NAFLD with figures ranging between 30 to 50% was reported in South American and Mexican populations^3^. In China, NAFLD is one of the leading etiologies of chronic liver diseases, with a prevalence ranging from 15.9%^4^ to 43.3%^5^ in the general population.

The pathogenesis of NAFLD is multifactorial with strong genetic and environmental contributions, such as unbalanced diets, over-nutrition, as well as sedentary lifestyles^6^. The potential effect of environmental endocrine disruptors (EEDs) on the increased incidence of NAFLD has raised a concern in recent years^7^. Nonylphenol (NP) is a well-known environmental endocrine disrupting chemical with weak estrogenic activity^8^, which is used in detergents, emulsifiers, and wetting agents in industry, but is also found in paints, pesticides, and household toiletries^9^. NP has been detected in human urine samples^10^ and adipose tissues^11^. Studies have suggested that NP has deleterious effects on endocrine^12^, reproductive^13^, immune^14^, and nervous^15,16^ systems in animals and humans.

Recent studies have indicated that exposure to EEDs is associated with an increased risk of metabolic disorders^17,18^, such as steatosis^19^, obesity^20,21^, insulin resistance^22^, and type 2 diabetes^23^. Wei *et al*. have demonstrated that bisphenol A exposure during the perinatal period exacerbated non-alcoholic steatohepatitis-like phenotype in high-fat diet (HFD)–treated male rat offspring^24^. In another study, Boucher et al. have found that polychlorinated biphenyls (PCB) interfere with the normal functioning of vital metabolic pathways in the liver and are associated with the development of NAFLD^25^. In addition, in our previous study, histopathological study in the liver revealed steatosis with hepatocellular ballooning degeneration in rats exposed to HSHFD plus NP^26^. Nonetheless, the effect of NP on the promotion of NAFLD development has not been demonstrated yet. Therefore, the present study was designed to test the hypothesis that subchronic NP exposure causes a predisposition to NAFLD. Specifically, we tested whether a combination of NP and high-sucrose/high-fat diet interacts in an additive or an antagonistic way.

## Materials and Methods

### Ethics Statement

Animal experimental procedures were approved by Zunyi Medical University Ethics Committee. All methods were performed in accordance with guidelines and regulations of the Zunyi Medical University.

### Animals and experimental protocol

Fifty Sprague-Dawley (SD) rats were obtained from the Animal Center of the Third Military Medical University (Chongqing, China), and received chow diet for 4 weeks. The rats (half males and half females) were randomly divided into 5 groups, each group had 10 rats. The rats were treated for 90 days. The first group fed with a normal diet (SCXK 2012–0012) (C-ND). The rats gavaged with groundnut oil alone (2 ml/kg/day). The second group fed with a high-sucrose/high-fat diet (SCXK2012–0012) (C-HSHFD). The third, fourth, and fifth groups were fed high-sucrose/high-fat diets with increasing concentrations of NP (Tokyo Chemical Co., Ltd, Tokyo, Japan, purity > 99%), which was dissolved in groundnut oil, at dose levels of 0.02 μg/kg/day (NP-L-HSHFD), 0.2 μg/kg/day (NP-M-HSHFD), or 2 μg/kg/day (NP-H-HSHFD). The ingredient of HSHFD was lard (15%), sugar (25%) and a normal diet (65%). The rats were raised under controlled temperature (20 ± 1 °C) and humidity (60 ± 5%), on a 12 – hr light (09:00–21:00 hr), 12 – hr dark (21:00–09:00 hr) cycle. Food and water were unlimitedly supplied to the animals.

### Measurement of body weight and hepatosomatic index (HSI)

The body weight of rats was measured on day 90. The HSI was calculated as the ratio of the liver weight to the total body weight (HSI = liver weight/total body weight × 100).

### Detection of liver oblique diameter

On day 80, Doppler color ultrasound (Philips Ultrasound, CA, USA) was used to detect the liver oblique diameter of rat and identify the rats with fatty liver^27^.

### Biochemical measurement

Pentobarbital sodium at a dose of 20 mg/kg was intraperitoneally injected to rats to induce anesthesia. Blood serum was gathered from the abdominal aorta for biochemical studies on day 90. Plasma alanine aminotransferase (ALT), aspartate aminotransferase (AST), total cholesterol (TC), triglyceride (TG), and low density lipoprotein cholesterol (LDL-C) activities were measured using a biochemical kit with an automatic biochemical analyzer (Beckman Coulter, Villepinte, France).

### Histopathology

The rats were euthanized by cardiac arrests, and liver tissues were gathered on day 90. Liver specimens were fixed in 10% formalin and stained with hematoxylin-eosin (HE) to detect hepatic steatosis and inflammation^28^.

### Detection of fatty acid synthase (FAS), sterol regulatory element-binding protein 1 (SREBP1) and uncoupling protein 2 (UCP2) mRNA in liver tissue by RT-PCR

Total RNA of lung tissue was extracted using TRIzol (Invitrogen, Diego, CA, USA). Reverse transcriptase was from Invitrogen (Diego, CA, USA), thermal cycler was from Biometra (Goettingen, Germany, model: T1-Thermob lock), and electrophoresis apparatus and gel imager were from Bio-Rad (Hercules, CA, USA). The levels of FAS, SREBP1, and UCP2 were expressed relative to glyceraldehyde-3-phosphate dehydrogenase (GAPDH)^29^. The sequences of the primers used are listed in Table 1.

**Table 1:** The sequences of the primers.

Gene name	Sequences	Gene ID
FAS	Forward:CTCTGGAAGTGCATGCTGTAAGA	246097
	Reverse:GGTAGATGTCATTTGCGAAAGGT	
SREBP1	Forward:CATCGACTACATCCGCTTCTTACA	78968
	Reverse:GTCTTTCAGTGATTTGATTTTGTGA	
UCP2	Forward:TGTGGTAAAGGTCCGCTTCC	54315
	Reverse:TTCGGGCAACATTGGGAG	
*β-action*	Forward:CAACGGCTCCGGCATGTGC	0311442
	Reverse:CTCTTGCTCTGGGCCTCG	

### Detection of peroxisome proliferator-activated receptor γ (PPARγ) and CCAAT/enhancer-binding protein α (C/EBPα) in liver tissue by western blotting

Western blotting was performed according to standard procedures. Briefly, cells were lysed in a lysis buffer containing 50 mM Tris-HCl (pH 8.0), 0.4% Nonidet P - 40, 120 mM NaCl, 1.5 mM MgCl_2_, 0.1% sodium dodecyl sulfate (SDS), 2 mM phenylmethylsulfonyl fluoride, 80 µg/mL leupeptin, 3 mM NaF and 1 mM dithiothreitol (DTT). Cell lysates (50 µg protein) were separated by 10% SDS-polyacrylamide gel electrophoresis, transferred onto a polyvinylidene fluoride membrane, blocked with 5% skim milk, and incubated with primary antibodies. PPARγ and C/EBPα antibodies were from Abcam (Cambridge, MA, USA) and the monoclonal β-actin antibody was from Chemicon (Temecula, California, USA). Horseradish peroxidase (HRP)-labelled mouse anti-rabbit IgG was from Jackson ImmunoResearch (West Grove, PA, USA). After incubation with HRP-conjugated secondary antibody at room temperature, immunoreactive proteins were detected using a chemiluminescent ECL Assay Kit (Amersham Pharmacia, Little Chalfont, England, UK) according to the manufacturer’s instructions^30^.

### Statistical analysis

SPSS18.0 packed programs for Windows (SPSS Inc., Chicago, IL, USA) was used to perform all calculations. Data are expressed as mean ± standard deviation. The One-way analysis of variance (ANOVA) test and Student–Newman–Keuls (SNK) test were used for the evaluation of differences among the treatment groups. A P-value < 0.05 among groups was considered as the level of statistical significance.

## Results

### Body weight and HSI

**Figure 1:**
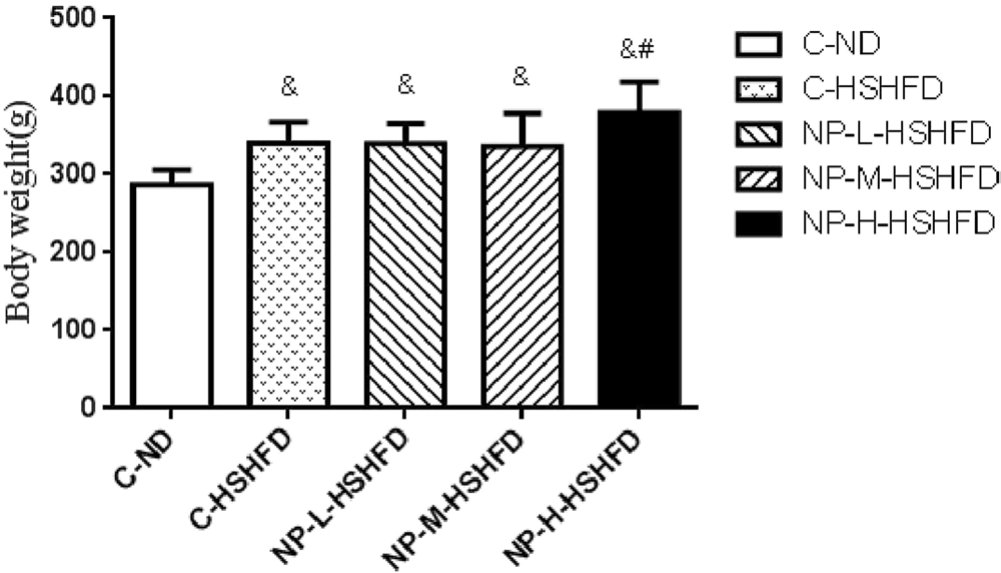
Difference of the body weight of rats among treatment groups (n = 10). ^&^*p* < 0.05 vs C-ND; ^#^*p* < 0.05 vs C-HSHFD.

The rats treated with HSHFD (C-HSHFD, NP-L-HSHFD, NP-M-HSHFD and NP-H-HSHFD) for 90 days had significantly increased body weight in comparison with control C-ND group (F = 10.477, *p* = 4.39E-6). The weight gain in the NP-H-HSHFD group was higher than that in the C-HSHFD group (*p* = 0.009; Fig. 1).

**Figure 2:**
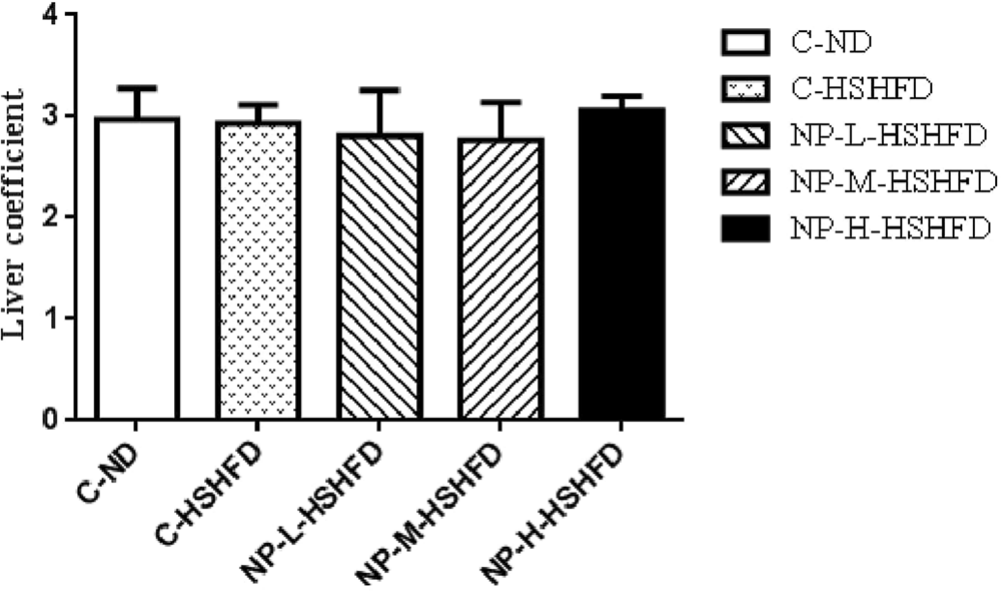
Difference of hepatosomatic index among treatment groups (n = 10).

There was no significant difference in the hepatosomatic index (HSI) between the individual groups (*p* > 0.05; Fig. 2).

### Daily volume of water consumed

**Figure 3:**
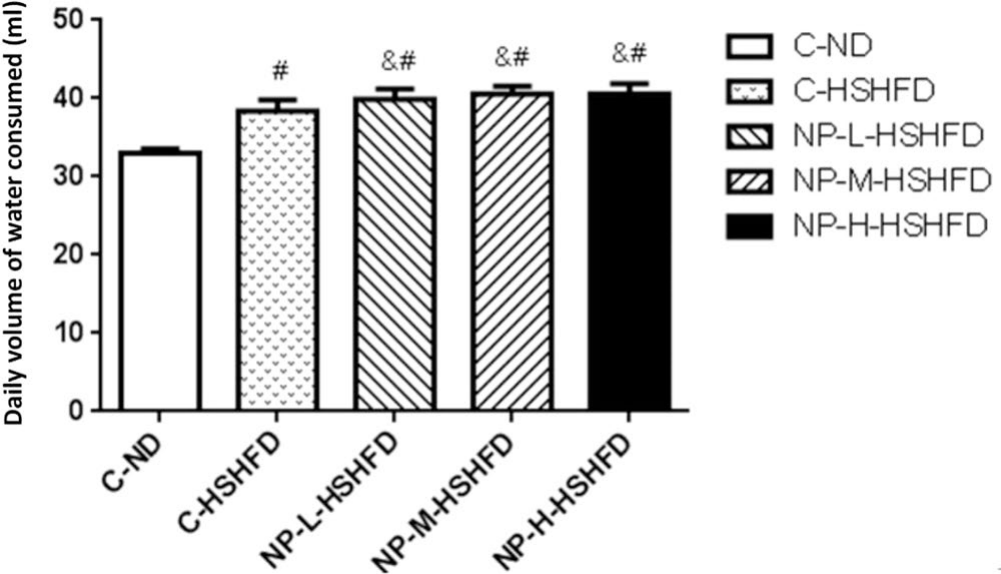
Comparison in the daily volume of water consumed among treatment groups (n = 10). ^#^*p* < 0.05 vs C-ND; ^&^*p* < 0.05 vs C-HSHFD.

Exposure to C-HSHFD, NP-L-HSHFD, NP-M-HSHFD and NP-H-HSHFD induced an increase in the daily volume of water consumed, as compared to the C-ND group (F = 80.61, *p = *6.27E-20). Exposure to NP-L-HSHFD, NP-M-HSHFD and NP-H-HSHFD significantly increased the daily volume of water consumed in comparison with the C-HSHFD group (*p* < 0.05; Fig. 3).

### Daily food intake

**Figure 4:**
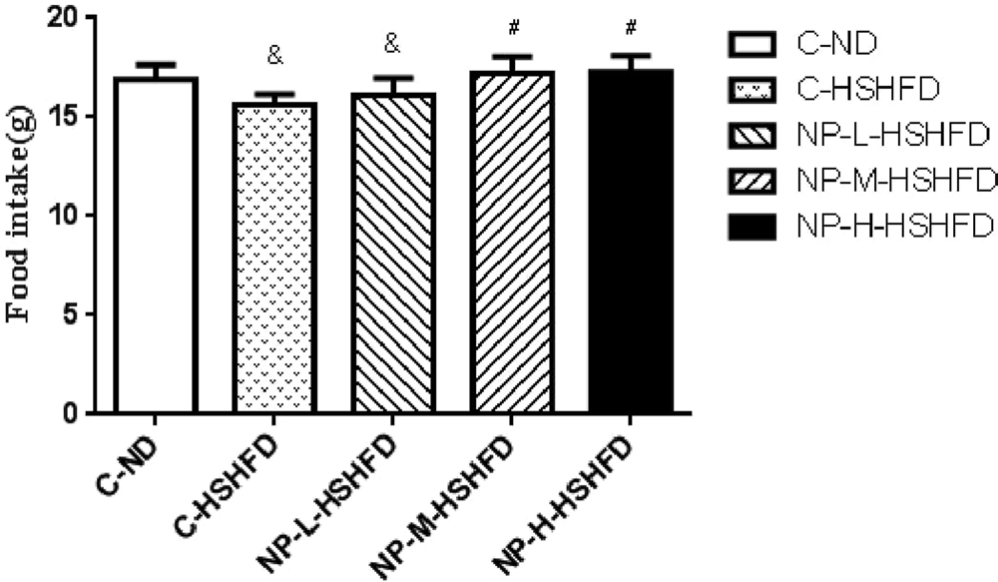
Comparison in the daily food intake among treatment groups (n = 10). ^&^*p* < 0.05 vs C-N; ^#^*p* < 0.05 vs C-HSHFD.

Exposure to C-HSHFD and NP-L-HSHFD decreased the daily food intake in comparison with the C-ND group (*p* < 0.05). However, NP-M-HSHFD and NP-H-HSHFD exposure increased the daily food intake in comparison with the C-HSHFD group (*p* < 0.05, Fig. 4).

### Ultrasound imaging

**Figure 5:**
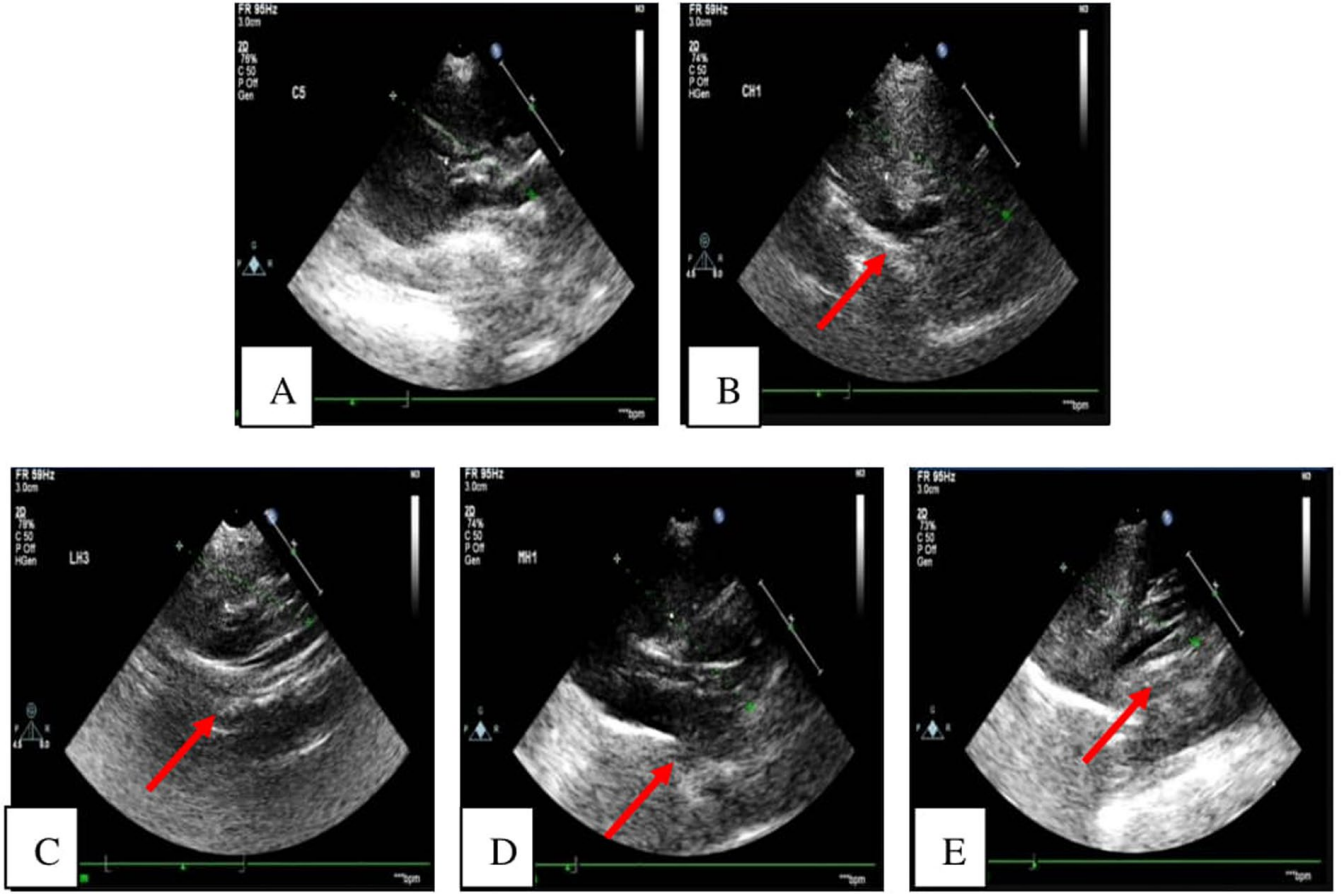
Ultrasound imaging of Liver. (**A**) C-ND group; (**B**) C-HSHFD group; (**C**) NP-L-HSHFD group; (**D**) NP-M-HSHFD group; E: NP-H-HSHFD group.

No obvious abnormal alterations were observed in the liver tissue of C-ND group. Increased hepatic echogenicity was observed in the C-HSHFD, NP-L-HSHFD, NP-M-HSHFD, and NP-H-HSHFD groups (arrowheads), which identified the rats with fatty liver. There was increased hepatic echogenicity in the NP-M-HSHFD and NP-H-HSHFD groups in comparison with the C-HSHFD group, indicating that NP-M-HSHFD and NP-H-HSHFD exposure results in worse fatty liver (Fig. 5).

**Figure 6:**
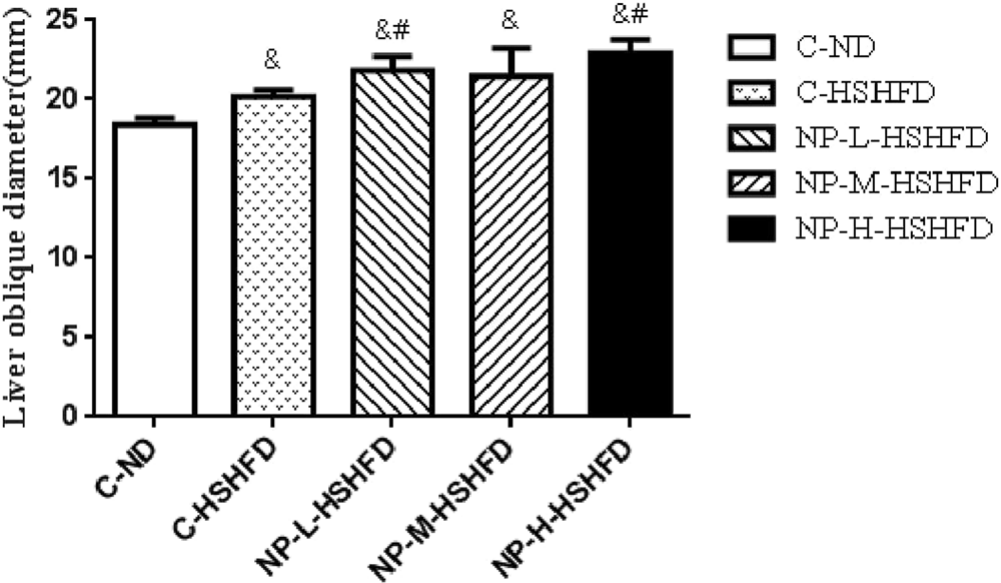
Difference of liver oblique diameter among treatment groups (n = 10). ^&^*p* < 0.05 vs C-ND; ^#^*p* < 0.05 vs C-HSHFD.

Liver oblique diameter in the NP-M-HSHFD (21.80 ± 1.47 mm) and NP-H-HSHFD (22.12 ± 2.13 mm) groups rather than NP-L-HSHFD (21.15 ± 1.69 mm) was significantly larger than that in the C-HSHFD (20.35 ± 0.47 mm) group (*p < *0.05). Liver oblique diameter in the C-ND group (18.62 ± 0.45 mm) was smaller than that in the NP-L-HSHFD, NP-M-HSHFD and NP-H-HSHFD groups (*p* < 0.05; Fig. 6).

### Plasma markers

**Figure 7:**
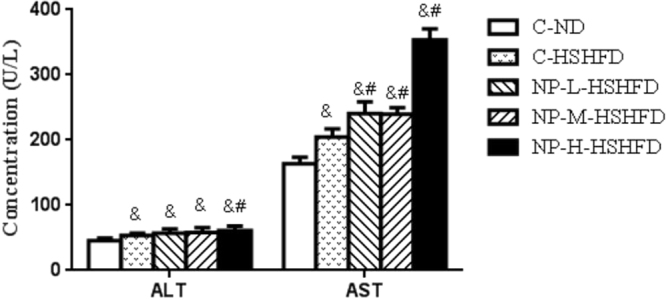
Comparisons in ALT and AST among treatment groups (n = 10). ^&^*p* < 0.05 vs C-ND; ^#^*p* < 0.05 vs C-HSHFD.

**Figure 8:**
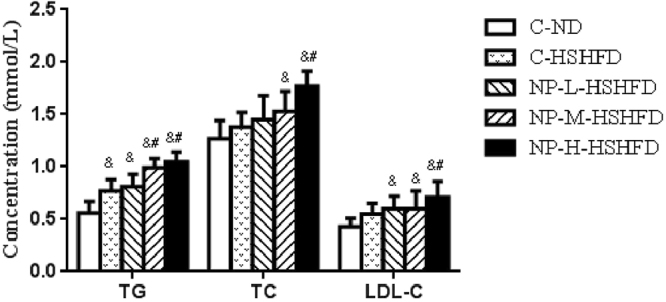
Comparisons in TG, TC and LDL-C among treatment groups (n = 10). ^&^*p* < 0.05 vs C-ND; ^#^*p* < 0.05 vs C-HSHFD.

Exposure to C-HSHFD, NP-L-HSHFD, NP-M-HSHFD and NP-H-HSHFD increased plasma ALT and AST levels in comparison with the C-ND group (*p* < 0.05). Exposure to NP-H-HSHFD increased plasma ALT and AST levels in comparison with the C-HSHFD group (*p* < 0.05; Fig. 7). Exposure to C-HSHFD, NP-L-HSHFD, NP-M-HSHFD, and NP-H-HSHFD increased plasma TC, TG, and LDL-C concentrations in comparison with the C-ND group. NP-H-HSHFD exposure increased plasma TC, TG and LDL-C levels in comparison with the C-HSHFD group (*p* < 0.05; Fig. 8).

### Histopathological changes in liver tissue

The hepatic cells in the C-ND group had a normal morphology. The appearance of hepatocytes, central vein, and portal areas appeared normal. Further, there was no evidence of inflammatory cell infiltration.

**Figure 9:**
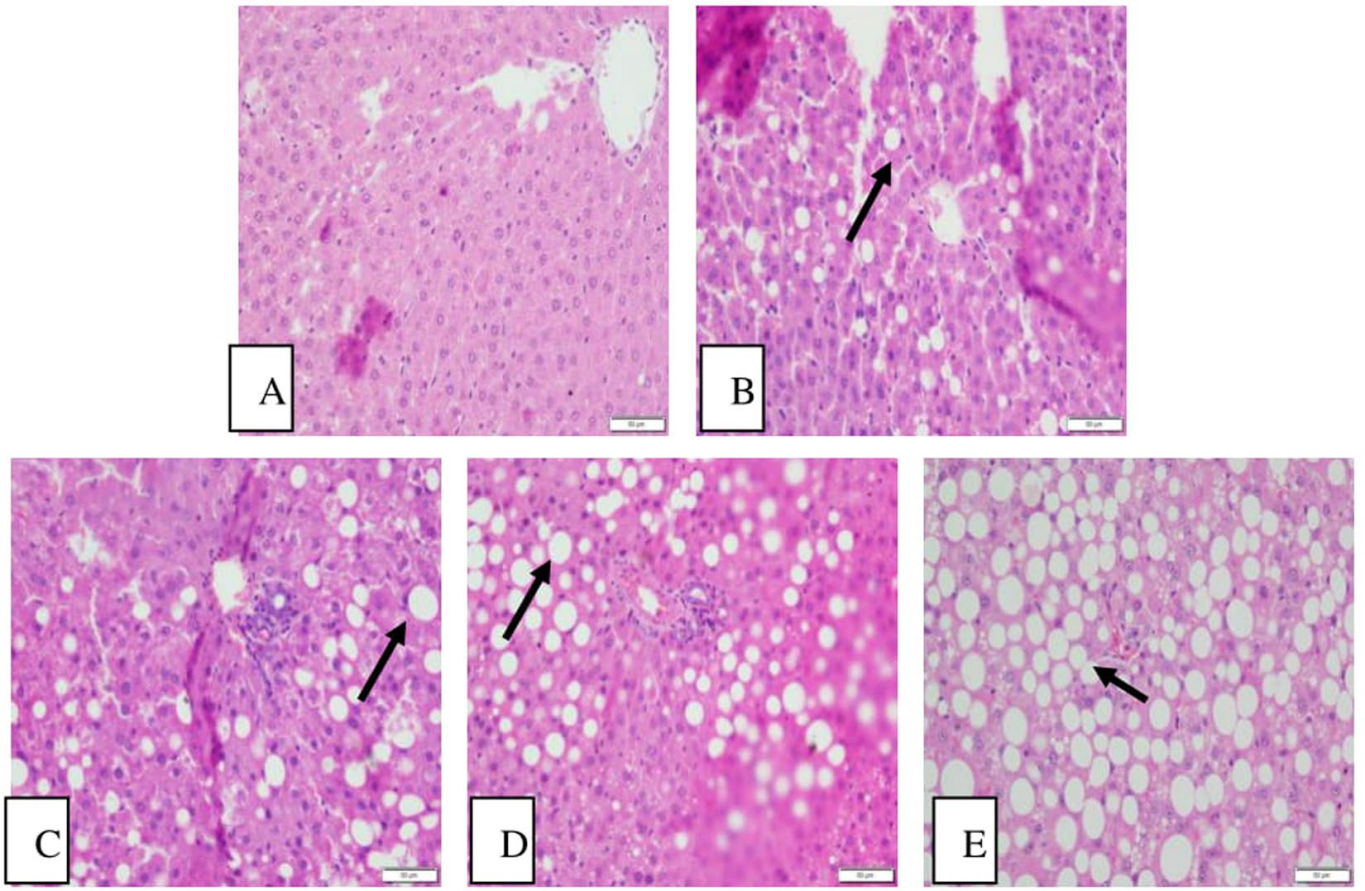
Histopathological results in the liver (200×). (**A**) C-ND group; (**B**) C-HSHFD group; (**C**) NP-L-HSHFD group; (**D**) NP-M-HSHFD group; (**E**) NP-H-HSHFD group.

Exposure to NP-H-HSHFD induced macro-vesicular steatosis (arrowheads). There were the accumulation of lipid droplets in the cytoplasm, dilation and congestion of central vein, and inflammatory cell infiltration in the NP-H-HSHFD group (Fig. 9).

### Effect of NP and HSHFD on gene expression of SREBP1, FAS, and UCP2 in liver

**Figure 10:**
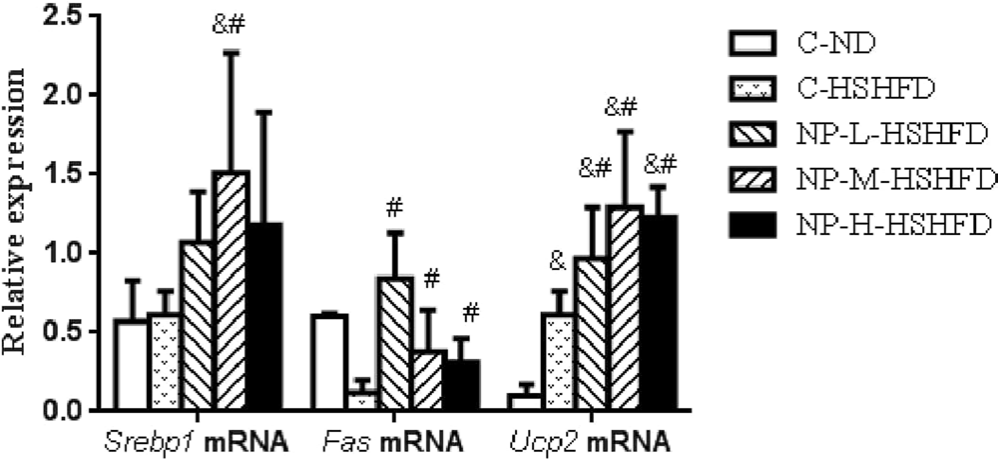
Expressions of *FAS*, *SREBP1* and *UCP2*mRNA in liver (n = 10). ^&^*p* < 0.05 vs C-ND; ^#^*p* < 0.05 vs C-HSHFD.

Compared to the control C-ND and C-HSHFD groups, expression of liver SREBP1 mRNA was increased in the groups fed with NP, even though it reached statistical significance only in the NP-M-HSHFD group (Fig. 10). Expression of FAS mRNA in the groups fed with NP was increased compared to the C-HSHFD group (*p* < 0.05). Similarly, expression of UCP2 mRNA was increased in the groups fed with NP compared to the C-HSHFD group (*p* < 0.05).

### Effect of NP and HSHFD on protein levels of C/EBPα and PPARγ in liver

**Figure 11:**
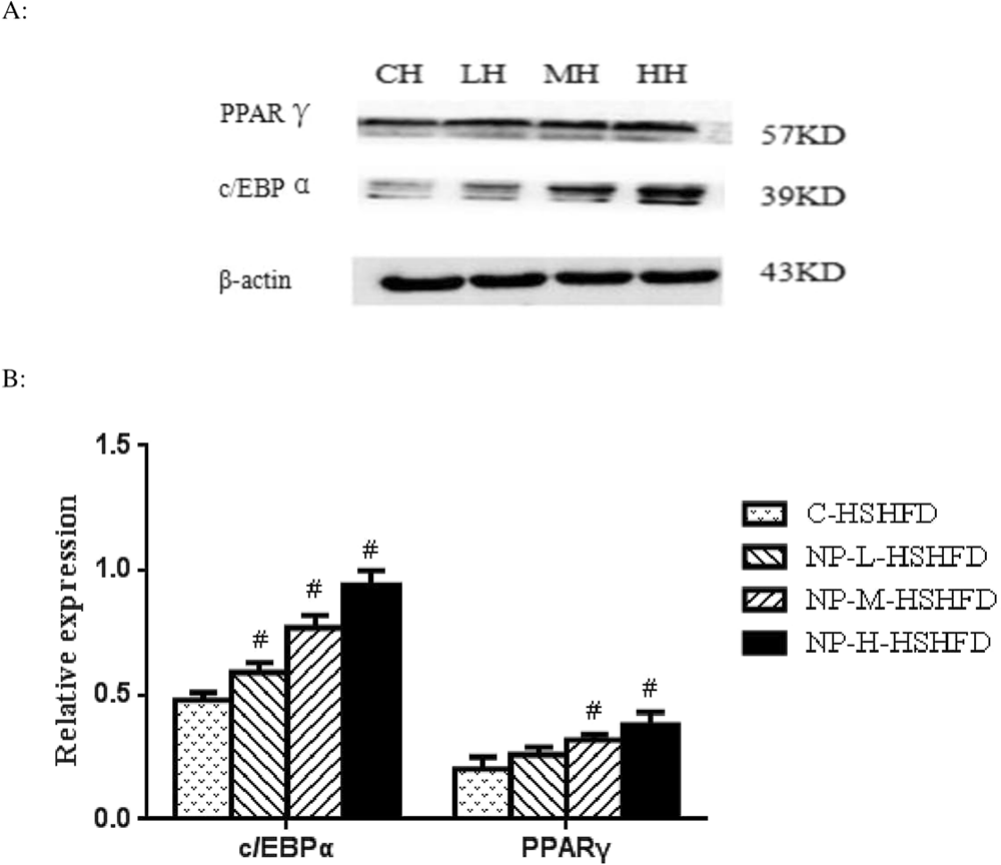
Effect of NP plus HSHFD on the levels of C/EBPα and PPARγ (n = 10). (**A**) Equal amounts of protein (50 µg) of cell lysate were analyzed by Western blotting to detect CCAAT enhancer-binding protein α (C/EBP α) and gamma receptors by peroxisome proliferators (PPAR γ) levels; (**B**) The graph of the protein relative expressions was obtained as describe in materials and methods. CH: C-HSHFD group; LH: NP-L-HSHFD group; MH: NP-M-HSHFD group; HH: NP-H-HSHFD group. ^#^*p* < 0.05 vs C-HSHFD.

The protein levels of C/EBPα were increased in the groups fed with NP in comparison with the C-HSHFD group (*p* < 0.05). PPARγ protein levels were increased only in the NP-M-HSHFD and NP-H-HSHFD groups compared to the C-HSHFD group (*p* < 0.05; Fig. 11).

## Discussion

In addition to genetic susceptibility, environmental factors, such as diet and lifestyle, play an important role in the pathogenesis of progressive NAFLD. Nonylphenol (NP) is a common environmental contaminant that is known to disrupt the endocrine system. NP can enter the body by ingestion or adsorption, and may mimic estrogenic action. Consequently, estrogen-like actions of NP have accounted for its adverse effects, such as reproductive malformations^31^, incapacity of offspring to react to certain behavioral stimuli^16^, and metabolic dysregulation^32^. However, until recently, little information on the metabolic effects of subchronic NP exposure was available. Therefore, in this study, we have investigated the effect of environmentally used concentrations of NP, together with HSHFD, on the pathogenesis of NAFLD.

Our results show that the combined exposure to NP and high sucrose-high fat diet results in metabolic disorders, including increased body weight, daily food intake and volume of water consumed, and hepatic echogenicity and liver oblique diameter, demonstrating that NP induces NAFLD in rats. In addition, our data demonstrate that HSHFD promoted the adverse effects of NP. Our data show hepatic steatosis with inflammatory cell infiltration in the NP-H-HSHFD group, indicating that a high-sucrose/high-fat diet combined with NP exposure could cause non-alcoholic steatohepatitis (NASH). Additionally, since the percentage of steatosis increased with the NP concentrations, these data indicate that the degree of NP exposure correlates with the degree of NAFLD steatosis in rats fed with HSHFD.

Increased serum ALT and AST levels serve as markers of the liver damage caused by EEDs^33^. The LDL is the major transport vehicle for TG in the blood. Our results showed that the administration of NP coupled with HSHFD increased the plasma levels of ALT, AST, TG, TC, and LDL-C, indicating that the exposure to NP + HSHFD causes a severe disturbance of lipid and protein metabolism. Our observations are in line with previous studies demonstrating the enhanced effect of other EEDs on lipid metabolism disorder in liver tissue^34,35^. Our study indicates that NP might regulate the lipid metabolism in adipocytes.

Our results also showed that exposure to NP + HSHFD increased expression of SREBP1, FAS, and UCP2 mRNA. SREBP1 is an important liver transcription factor that regulates lipid synthesis and enzymes (e.g., fatty acid synthase) catalyzing various steps in the FA and TG synthesis pathways^36,37^. SREBP-1c-activated lipogenesis has been suggested to contribute to NAFLD^38^. FAS catalyzes lipid synthesis in the cytosol^39^, while UCP2, an anion carrier, inhibits mitochondrial membrane potential and ATP synthesis^40^. Our results demonstrate that NP exposure increases expression of fat synthesis-related genes, suggesting that NP may increase the lipid levels via the upregulation of SREBP1, FAS and UCP2 gene expression in the liver. These data are consistent with a previous study, which demonstrated that gene expression of SREBP-1C and FAS was increased in liver of EEDs-exposed rats^41^.

PPARγ is predominantly expressed in adipose tissues, and is considered as the key regulator of adipocyte differentiation and adipogenesis^42^. C/EBPα is a key transcriptional regulator that plays an important role during adipocyte differentiation and function. A recent study has demonstrated that in 3T3-L1 adipocytes differentiated in the presence of BPA, the expression of PPARγ and C/EBPα was increased^43^. The current study found that NP-HSHFD combined exposure was associated with the increased expressions of PPARγ and C/EBPα, suggesting that NP induces pre-adipocyte differentiation through altering the expression of these transcription factors. These observations are in accordance with previous publications demonstrating increased expression of PPARγ proteins in the fat tissues of EEDs (i.e., tributyltin chloride (TBT))-treated rats^44^. Importantly, our findings provide the first evidence of altered gene expression in the liver after combined exposure to NP and high sucrose-high fat diet.

The unit of NP exposure dose in prior studies was milligram (mg.)^15,45^, which was far above actual environmental exposure level. For example, NP exposure doses changed from 50 mg/kg/day to 200 mg/kg/day in our toxicological studies^46,47^. Specifically, our previous study shown that the concentration of NP measured in Xiangjiang River in Zunyi of China ranges from 0.174 to 3.411 μg/L^48^. In light of the findings, the NP gavage doses of 0.02 μg/kg - 2 μg/kg in rats were designed in this study. In addition, exposure time for NP were short-term acute or subacute period in previous studies^43,49^, which was far shorter than the actual exposure time. In order to make up the above shortcomings, the current study designed low environmental exposure concentration and subchronic exposure time, respectively.

The objective of our prior study^27^ was to explore qualitatively whether NP exposure might induce NAFLD, therefore, only single NP exposure dose (180 mg/kg/day) was designed, and the unit of NP exposure dose was milligram. The current study is the extension and expansion of the prior study, which has two main differences from the former one: 1. the design of exposure dose. In the current study, NP exposure dose was close to human exposure level, which is microgramme level, and three levels of NP treatment dose were designed to observe a dose-effect relationship. 2. The exploration of mechanism. The detection indicators of lipid metabolism-related genes and proteins in liver were added on the basis of the former study.

In conclusion, the present study demonstrates that prolonged exposure to low NP dose coupled with a HSHFD aggravates NAFLD in rats, as evidenced by increased body weight, increased daily volume of water consumed and daily food intake, increased hepatic echogenicity and oblique diameter of the liver, and increased plasma levels of ALT, AST, TC, TG and LDL-C. Moreover, exposure to NP induces inflammatory cell infiltration in the liver, and increases expression of SREBP1, FAS, and UCP2 in hepatocytes. Importantly, HSHFD promoted the adverse effects of NP. Together, our study indicates that subchronic NP exposure may lead to NAFLD, especially when combined with high-sucrose/high-fat diet.
